# 2-[(Diphenyl­phosphor­yl)(hy­droxy)meth­yl]-5-meth­oxy­phenol

**DOI:** 10.1107/S1600536812020685

**Published:** 2012-05-12

**Authors:** Yutian Shao, Chao Yang, Wujiong Xia

**Affiliations:** a State Key Laboratory of Urban Water Resource and Environment (SKLUWRE) & Academy of Fundamental and Interdisciplinary Sciences, Harbin Institute of Technology, Harbin, Heilongjiang 150090, People’s Republic of China

## Abstract

In the title compound, C_20_H_19_O_4_P, the dihedral angle between the phenyl rings is 73.3 (4)° and the dihedral angles between the benzene ring and the two phenyl rings are 43.0 (3) and 54.3 (1)°. In the crystal, O—H⋯O hydrogen bonds and weak O—H⋯O inter­actions are observed, which form a supra­molecular sheet parallel to (010).

## Related literature

For α-hy­droxy­lphosphine oxides, see: Marmor & Seyferth (1969 ▶); Toyota *et al.* (1993 ▶); Kaza­nkova *et al.* (2003 ▶); For substrates used in the preparation of α-carboxyl­phosphine oxides, see: Fischer *et al.* (1993 ▶) and for substrates used in the preparation of unsymmetrical phosphine oxides, see: Miller *et al.* (1957 ▶).
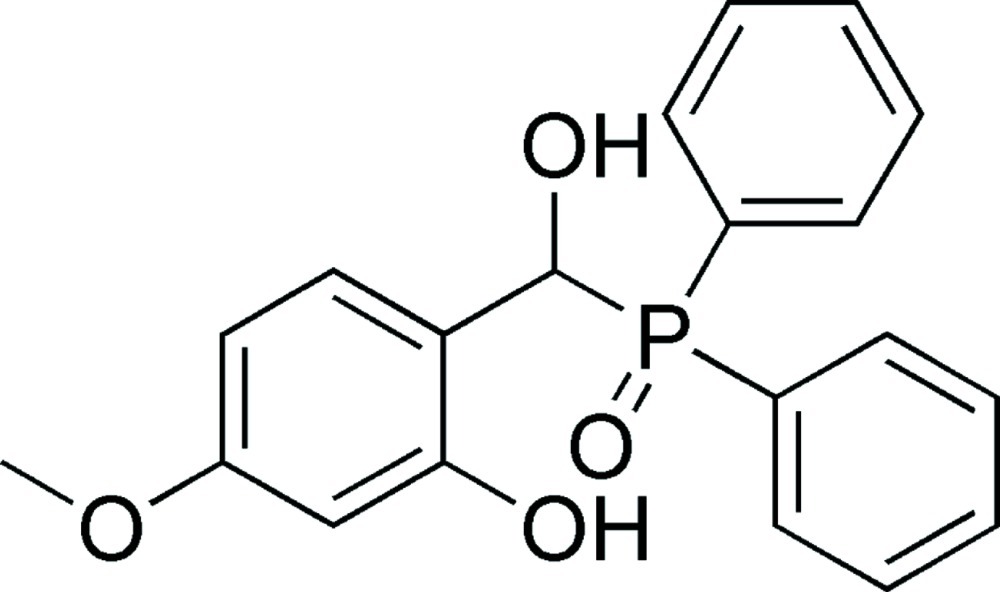


## Experimental

### 

#### Crystal data

C_20_H_19_O_4_P
*M* *_r_* = 354.32
Monoclinic, 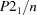

*a* = 8.349 (7) Å
*b* = 17.406 (14) Å
*c* = 12.639 (10) Å
β = 107.863 (9)°
*V* = 1748 (2) Å^3^
*Z* = 4
Mo *K*α radiation
μ = 0.18 mm^−1^
*T* = 296 K
0.20 × 0.15 × 0.10 mm

#### Data collection

Bruker SMART CCD APEXII diffractometer
Absorption correction: multi-scan (*SADABS*; Bruker, 2004 ▶) *T*_min_ = 0.965, *T*_max_ = 0.982
8150 measured reflections
3017 independent reflections
2124 reflections with *I* > 2σ(*I*)
*R*_int_ = 0.043

#### Refinement

*R*[*F* ^2^ > 2σ(*F* ^2^)] = 0.044
*wR*(*F* ^2^) = 0.114
*S* = 1.04
3017 reflections
229 parameters
H-atom parameters constrained
Δρ_max_ = 0.28 e Å^−3^
Δρ_min_ = −0.25 e Å^−3^

### 

Data collection: *APEX2* (Bruker, 2004 ▶); cell refinement: *SAINT* (Bruker, 2004 ▶); data reduction: *SAINT*; program(s) used to solve structure: *SHELXS97* (Sheldrick, 2008 ▶); program(s) used to refine structure: *SHELXL97* (Sheldrick, 2008 ▶); molecular graphics: *DIAMOND* (Brandenburg, 1999 ▶); software used to prepare material for publication: *SHELXL97*.

## Supplementary Material

Crystal structure: contains datablock(s) I, global. DOI: 10.1107/S1600536812020685/jj2135sup1.cif

Structure factors: contains datablock(s) I. DOI: 10.1107/S1600536812020685/jj2135Isup2.hkl

Supplementary material file. DOI: 10.1107/S1600536812020685/jj2135Isup3.cml

Additional supplementary materials:  crystallographic information; 3D view; checkCIF report

## Figures and Tables

**Table 1:** Hydrogen-bond geometry (Å, °)

*D*—H⋯*A*	*D*—H	H⋯*A*	*D*⋯*A*	*D*—H⋯*A*
O2—H2⋯O1^i^	0.82	1.81	2.613 (2)	168
O4—H4⋯O3^ii^	0.82	2.28	3.046 (3)	156

Symmetry codes: (i) 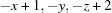; (ii) 
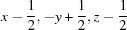
.

**Fig. 1.**
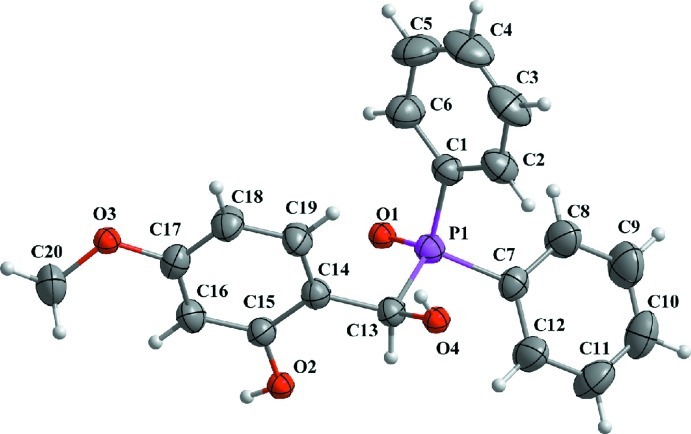
Molecular structure of the title compound showing the atom labeling scheme and 50% probability displacement ellipsoids.

**Fig. 2.**
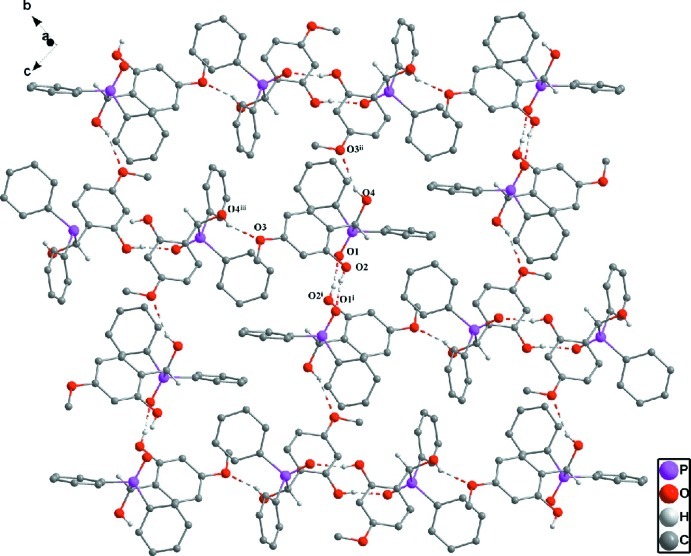
Packing diagram of the title compound viewed aloing the a axis. Dashed lines represent O—H···O hydrogen bonds and weak O—H···O intermolecular interactions. H atoms not involved in hydrogen bonding are omitted for clarity. [symmetry codes: (i) 1 - x, - y, 2 -z; (ii) x - 1/2, 0.5 - y, z - 0.5; (iii) x + 1/2, 0.5 - y, 0.5 + z].

## supplementary crystallographic information

### Comment

α-hydroxylphosphine oxides are molecules (Marmor *et al.*, 1969; Toyota, *et al.*, 1993; Kazankova *et al.*., 2003) that are used as substrates for the preparation of α-carboxylphosphine oxides (Fischer, *et al.*, 1993) and unsymmetrical phosphine oxides (Miller *et al.*, 1957). We present herin, the preparation and crystal structure of the title compound, C_20_H_19_O_4_P, (I).

In the title compound (I), the dihedral angle between the two mono-substituted benzene rings is 73.3 (4)° (Fig. 1). The dihedral angle between the tri- substituted benzene ring and two mono-substituted benzene rings is 43. 0(3)° and 54.3 (0)°, respectively. O—H···O hydrogen bonds and weak O—H···O intermolecular interactions (Table 1) are observed which form a two-dimensional supramolecular sheet and influence crystal packing (Fig. 2).

### Experimental

The title compound was obtained from the following procedure. To a flame dried round-bottomed flask, 2-hydroxy-4-methoxy-benzaldehyde (1.0equiv), potassiumtert-butoxide (1.5equiv) and anhydrous DMF were added under N_2_ protection. After the addition of chlorodiphenylphosphine (1.5 equiv. in anhydrous DMF) and stirred overnight at room temperature, water was added to quench the reaction. The product was extracted with CH_2_Cl_2_, dried with Na_2_SO_4 _and concentrated under pressure to give an oil residue, which was purified through a silica gel column to yield the title compound.

### Refinement

All H atoms were placed in calculated positions and then refined using the riding model, with atom–H lengths of 0.93Å (CH), 0.98Å or 0.82Å (OH) Isotropic displacement parameters were set to 1.2 (CH) or 1.5 (CH_3_) times *U*_eq_ of the parent atom.

### Crystal data

**Table:**

C_20_H_19_O_4_P	*F*(000) = 744
*M**_r_* = 354.32	*D*_x_ = 1.346 Mg m^−^^3^
Monoclinic, *P*2_1_/*n*	Mo *K*α radiation, λ = 0.71073 Å
Hall symbol: -P 2yn	Cell parameters from 255 reflections
*a* = 8.349 (7) Å	θ = 25.6–3.1°
*b* = 17.406 (14) Å	µ = 0.18 mm^−^^1^
*c* = 12.639 (10) Å	*T* = 296 K
β = 107.863 (9)°	Block, colourless
*V* = 1748 (2) Å^3^	0.20 × 0.15 × 0.10 mm
*Z* = 4	

### Data collection

**Table:**

Bruker SMART CCD APEXII diffractometer	3017 independent reflections
Radiation source: fine-focus sealed tube	2124 reflections with *I* > 2σ(*I*)
Graphite monochromator	*R*_int_ = 0.043
Detector resolution: 10 pixels mm^-1^	θ_max_ = 25.1°, θ_min_ = 2.1°
ω scans	*h* = −9→7
Absorption correction: multi-scan (*SADABS*; Bruker, 2004)	*k* = −20→20
*T*_min_ = 0.965, *T*_max_ = 0.982	*l* = −15→12
8150 measured reflections	

### Refinement

**Table:**

Refinement on *F*^2^	Primary atom site location: structure-invariant direct methods
Least-squares matrix: full	Secondary atom site location: difference Fourier map
*R*[*F*^2^ > 2σ(*F*^2^)] = 0.044	Hydrogen site location: inferred from neighbouring sites
*wR*(*F*^2^) = 0.114	H-atom parameters constrained
*S* = 1.04	*w* = 1/[σ^2^(*F*_o_^2^) + (0.0511*P*)^2^ + 0.1885*P*] where *P* = (*F*_o_^2^ + 2*F*_c_^2^)/3
3017 reflections	(Δ/σ)_max_ < 0.001
229 parameters	Δρ_max_ = 0.28 e Å^−^^3^
0 restraints	Δρ_min_ = −0.25 e Å^−^^3^

### Special details

**Table:**

Geometry. All e.s.d.'s (except the e.s.d. in the dihedral angle between two l.s. planes) are estimated using the full covariance matrix. The cell e.s.d.'s are taken into account individually in the estimation of e.s.d.'s in distances, angles and torsion angles; correlations between e.s.d.'s in cell parameters are only used when they are defined by crystal symmetry. An approximate (isotropic) treatment of cell e.s.d.'s is used for estimating e.s.d.'s involving l.s. planes.
Refinement. Refinement of *F*^2^ against ALL reflections. The weighted *R*-factor *wR* and goodness of fit *S* are based on *F*^2^, conventional *R*-factors *R* are based on *F*, with *F* set to zero for negative *F*^2^. The threshold expression of *F*^2^ > σ(*F*^2^) is used only for calculating *R*-factors(gt) *etc*. and is not relevant to the choice of reflections for refinement. *R*-factors based on *F*^2^ are statistically about twice as large as those based on *F*, and *R*- factors based on ALL data will be even larger.

### Fractional atomic coordinates and isotropic or equivalent isotropic displacement parameters (Å^2^)

**Table:**

	*x*	*y*	*z*	*U*_iso_*/*U*_eq_	
P1	0.18172 (8)	0.11354 (3)	0.82003 (5)	0.0358 (2)	
O1	0.2141 (2)	0.08777 (9)	0.93707 (13)	0.0448 (5)	
O2	0.6011 (2)	0.00424 (8)	0.91075 (14)	0.0510 (5)	
H2	0.6570	−0.0200	0.9652	0.077*	
O3	0.9492 (2)	0.21531 (10)	1.09662 (15)	0.0536 (5)	
O4	0.3359 (2)	0.13072 (10)	0.66335 (14)	0.0510 (5)	
H4	0.3793	0.1733	0.6665	0.077*	
C1	0.1266 (3)	0.21306 (13)	0.8032 (2)	0.0380 (6)	
C2	0.0631 (3)	0.24707 (15)	0.6995 (2)	0.0497 (7)	
H2A	0.0431	0.2171	0.6359	0.060*	
C3	0.0295 (4)	0.32424 (16)	0.6896 (3)	0.0639 (9)	
H3	−0.0112	0.3466	0.6196	0.077*	
C4	0.0560 (4)	0.36781 (16)	0.7825 (3)	0.0725 (10)	
H4A	0.0307	0.4200	0.7757	0.087*	
C5	0.1198 (5)	0.33591 (16)	0.8866 (3)	0.0749 (10)	
H5	0.1385	0.3665	0.9495	0.090*	
C6	0.1560 (4)	0.25804 (15)	0.8973 (2)	0.0551 (8)	
H6	0.1998	0.2362	0.9675	0.066*	
C7	0.0125 (3)	0.06133 (13)	0.7244 (2)	0.0389 (6)	
C8	−0.1523 (4)	0.07782 (17)	0.7203 (2)	0.0587 (8)	
H8	−0.1737	0.1176	0.7632	0.070*	
C9	−0.2837 (4)	0.03577 (19)	0.6533 (3)	0.0698 (9)	
H9	−0.3933	0.0477	0.6511	0.084*	
C10	−0.2562 (4)	−0.02306 (18)	0.5903 (3)	0.0629 (9)	
H10	−0.3460	−0.0517	0.5462	0.076*	
C11	−0.0960 (4)	−0.03961 (17)	0.5925 (3)	0.0651 (9)	
H11	−0.0764	−0.0792	0.5486	0.078*	
C12	0.0377 (4)	0.00201 (15)	0.6594 (2)	0.0572 (8)	
H12	0.1466	−0.0103	0.6605	0.069*	
C13	0.3672 (3)	0.10114 (13)	0.77302 (19)	0.0368 (6)	
H13	0.3831	0.0456	0.7680	0.044*	
C14	0.5235 (3)	0.13105 (13)	0.85807 (19)	0.0363 (6)	
C15	0.6364 (3)	0.07990 (12)	0.92673 (19)	0.0363 (6)	
C16	0.7810 (3)	0.10582 (13)	1.0067 (2)	0.0404 (6)	
H16	0.8560	0.0710	1.0519	0.048*	
C17	0.8119 (3)	0.18334 (14)	1.0182 (2)	0.0412 (6)	
C18	0.7035 (3)	0.23562 (14)	0.9503 (2)	0.0465 (7)	
H18	0.7269	0.2879	0.9575	0.056*	
C19	0.5600 (3)	0.20922 (13)	0.8717 (2)	0.0438 (7)	
H19	0.4860	0.2444	0.8268	0.053*	
C20	1.0787 (4)	0.16408 (17)	1.1566 (2)	0.0635 (9)	
H20A	1.0368	0.1320	1.2040	0.095*	
H20B	1.1732	0.1932	1.2009	0.095*	
H20C	1.1132	0.1326	1.1051	0.095*	

### Atomic displacement parameters (Å^2^)

**Table:**

	*U*^11^	*U*^22^	*U*^33^	*U*^12^	*U*^13^	*U*^23^
P1	0.0395 (4)	0.0306 (3)	0.0328 (4)	0.0029 (3)	0.0043 (3)	0.0020 (3)
O1	0.0551 (12)	0.0408 (9)	0.0339 (10)	0.0076 (8)	0.0070 (8)	0.0076 (8)
O2	0.0608 (13)	0.0318 (9)	0.0454 (12)	0.0020 (8)	−0.0059 (9)	0.0032 (8)
O3	0.0452 (11)	0.0561 (11)	0.0473 (11)	−0.0069 (9)	−0.0035 (9)	−0.0058 (9)
O4	0.0523 (12)	0.0566 (12)	0.0382 (11)	−0.0021 (9)	0.0052 (9)	−0.0001 (8)
C1	0.0376 (15)	0.0336 (12)	0.0420 (15)	0.0032 (10)	0.0113 (12)	0.0028 (11)
C2	0.0507 (17)	0.0484 (15)	0.0498 (17)	0.0114 (13)	0.0153 (14)	0.0107 (13)
C3	0.061 (2)	0.0520 (18)	0.082 (2)	0.0177 (15)	0.0261 (18)	0.0295 (17)
C4	0.077 (2)	0.0345 (16)	0.111 (3)	0.0128 (15)	0.036 (2)	0.0162 (19)
C5	0.098 (3)	0.0414 (17)	0.084 (3)	0.0054 (17)	0.027 (2)	−0.0193 (16)
C6	0.067 (2)	0.0413 (15)	0.0536 (19)	0.0057 (14)	0.0141 (16)	−0.0002 (13)
C7	0.0437 (16)	0.0392 (13)	0.0317 (14)	−0.0021 (11)	0.0083 (12)	0.0027 (11)
C8	0.0465 (18)	0.0670 (19)	0.064 (2)	−0.0067 (14)	0.0193 (15)	−0.0151 (15)
C9	0.0446 (19)	0.085 (2)	0.076 (2)	−0.0130 (17)	0.0135 (17)	−0.0075 (19)
C10	0.055 (2)	0.073 (2)	0.054 (2)	−0.0242 (16)	0.0065 (16)	−0.0039 (16)
C11	0.072 (2)	0.0586 (18)	0.063 (2)	−0.0190 (17)	0.0185 (18)	−0.0223 (15)
C12	0.0478 (18)	0.0513 (17)	0.070 (2)	−0.0069 (13)	0.0144 (16)	−0.0182 (15)
C13	0.0390 (15)	0.0327 (12)	0.0340 (14)	0.0028 (10)	0.0042 (11)	−0.0002 (10)
C14	0.0371 (14)	0.0358 (13)	0.0336 (14)	0.0011 (10)	0.0074 (11)	0.0002 (10)
C15	0.0399 (15)	0.0327 (13)	0.0337 (14)	0.0010 (11)	0.0073 (12)	−0.0022 (10)
C16	0.0407 (15)	0.0416 (14)	0.0355 (15)	0.0064 (11)	0.0065 (12)	0.0040 (11)
C17	0.0399 (15)	0.0464 (14)	0.0342 (15)	−0.0054 (12)	0.0070 (12)	−0.0039 (11)
C18	0.0501 (17)	0.0331 (13)	0.0487 (17)	−0.0054 (12)	0.0038 (14)	0.0000 (12)
C19	0.0463 (16)	0.0347 (13)	0.0420 (16)	0.0039 (11)	0.0012 (13)	0.0045 (11)
C20	0.0416 (18)	0.080 (2)	0.057 (2)	0.0037 (15)	−0.0022 (15)	−0.0090 (16)

### Geometric parameters (Å, º)

**Table:**

P1—O1	1.489 (2)	C8—C9	1.373 (4)
P1—C1	1.788 (3)	C8—H8	0.9300
P1—C7	1.799 (3)	C9—C10	1.359 (4)
P1—C13	1.833 (3)	C9—H9	0.9300
O2—C15	1.351 (3)	C10—C11	1.360 (4)
O2—H2	0.8200	C10—H10	0.9300
O3—C17	1.382 (3)	C11—C12	1.381 (4)
O3—C20	1.426 (3)	C11—H11	0.9300
O4—C13	1.426 (3)	C12—H12	0.9300
O4—H4	0.8200	C13—C14	1.507 (3)
C1—C6	1.382 (3)	C13—H13	0.9800
C1—C2	1.386 (3)	C14—C15	1.390 (3)
C2—C3	1.370 (4)	C14—C19	1.394 (3)
C2—H2A	0.9300	C15—C16	1.391 (3)
C3—C4	1.358 (4)	C16—C17	1.373 (3)
C3—H3	0.9300	C16—H16	0.9300
C4—C5	1.375 (4)	C17—C18	1.381 (3)
C4—H4A	0.9300	C18—C19	1.379 (3)
C5—C6	1.386 (4)	C18—H18	0.9300
C5—H5	0.9300	C19—H19	0.9300
C6—H6	0.9300	C20—H20A	0.9600
C7—C12	1.375 (4)	C20—H20B	0.9600
C7—C8	1.391 (4)	C20—H20C	0.9600
			
O1—P1—C1	111.82 (11)	C11—C10—H10	120.3
O1—P1—C7	112.41 (11)	C10—C11—C12	120.4 (3)
C1—P1—C7	106.81 (12)	C10—C11—H11	119.8
O1—P1—C13	111.88 (11)	C12—C11—H11	119.8
C1—P1—C13	106.70 (11)	C7—C12—C11	121.1 (3)
C7—P1—C13	106.86 (13)	C7—C12—H12	119.4
C15—O2—H2	109.5	C11—C12—H12	119.4
C17—O3—C20	117.1 (2)	O4—C13—C14	115.4 (2)
C13—O4—H4	109.5	O4—C13—P1	110.58 (16)
C6—C1—C2	119.2 (2)	C14—C13—P1	111.17 (17)
C6—C1—P1	118.36 (19)	O4—C13—H13	106.4
C2—C1—P1	122.41 (19)	C14—C13—H13	106.4
C3—C2—C1	120.9 (3)	P1—C13—H13	106.4
C3—C2—H2A	119.6	C15—C14—C19	117.9 (2)
C1—C2—H2A	119.6	C15—C14—C13	119.8 (2)
C4—C3—C2	119.6 (3)	C19—C14—C13	122.3 (2)
C4—C3—H3	120.2	O2—C15—C14	117.1 (2)
C2—C3—H3	120.2	O2—C15—C16	121.7 (2)
C3—C4—C5	120.9 (3)	C14—C15—C16	121.1 (2)
C3—C4—H4A	119.5	C17—C16—C15	119.3 (2)
C5—C4—H4A	119.5	C17—C16—H16	120.4
C4—C5—C6	119.8 (3)	C15—C16—H16	120.4
C4—C5—H5	120.1	C16—C17—C18	121.0 (2)
C6—C5—H5	120.1	C16—C17—O3	124.0 (2)
C1—C6—C5	119.6 (3)	C18—C17—O3	114.9 (2)
C1—C6—H6	120.2	C19—C18—C17	119.1 (2)
C5—C6—H6	120.2	C19—C18—H18	120.4
C12—C7—C8	117.6 (2)	C17—C18—H18	120.4
C12—C7—P1	123.2 (2)	C18—C19—C14	121.5 (2)
C8—C7—P1	119.0 (2)	C18—C19—H19	119.2
C9—C8—C7	120.4 (3)	C14—C19—H19	119.2
C9—C8—H8	119.8	O3—C20—H20A	109.5
C7—C8—H8	119.8	O3—C20—H20B	109.5
C10—C9—C8	121.1 (3)	H20A—C20—H20B	109.5
C10—C9—H9	119.5	O3—C20—H20C	109.5
C8—C9—H9	119.5	H20A—C20—H20C	109.5
C9—C10—C11	119.3 (3)	H20B—C20—H20C	109.5
C9—C10—H10	120.3		
			
O1—P1—C1—C6	14.3 (3)	C10—C11—C12—C7	−0.6 (5)
C7—P1—C1—C6	137.7 (2)	O1—P1—C13—O4	−176.33 (14)
C13—P1—C1—C6	−108.3 (2)	C1—P1—C13—O4	−53.73 (18)
O1—P1—C1—C2	−168.7 (2)	C7—P1—C13—O4	60.24 (18)
C7—P1—C1—C2	−45.3 (2)	O1—P1—C13—C14	−46.77 (19)
C13—P1—C1—C2	68.7 (2)	C1—P1—C13—C14	75.83 (18)
C6—C1—C2—C3	0.0 (4)	C7—P1—C13—C14	−170.20 (15)
P1—C1—C2—C3	−177.0 (2)	O4—C13—C14—C15	−130.8 (2)
C1—C2—C3—C4	−1.1 (4)	P1—C13—C14—C15	102.3 (2)
C2—C3—C4—C5	1.5 (5)	O4—C13—C14—C19	49.7 (3)
C3—C4—C5—C6	−0.7 (5)	P1—C13—C14—C19	−77.3 (3)
C2—C1—C6—C5	0.7 (4)	C19—C14—C15—O2	−178.5 (2)
P1—C1—C6—C5	177.8 (2)	C13—C14—C15—O2	1.9 (3)
C4—C5—C6—C1	−0.4 (5)	C19—C14—C15—C16	0.1 (4)
O1—P1—C7—C12	−100.7 (2)	C13—C14—C15—C16	−179.4 (2)
C1—P1—C7—C12	136.3 (2)	O2—C15—C16—C17	179.0 (2)
C13—P1—C7—C12	22.4 (3)	C14—C15—C16—C17	0.4 (4)
O1—P1—C7—C8	74.9 (2)	C15—C16—C17—C18	−1.3 (4)
C1—P1—C7—C8	−48.0 (2)	C15—C16—C17—O3	178.4 (2)
C13—P1—C7—C8	−161.9 (2)	C20—O3—C17—C16	10.2 (4)
C12—C7—C8—C9	0.0 (4)	C20—O3—C17—C18	−170.1 (2)
P1—C7—C8—C9	−175.9 (2)	C16—C17—C18—C19	1.7 (4)
C7—C8—C9—C10	0.5 (5)	O3—C17—C18—C19	−178.1 (2)
C8—C9—C10—C11	−1.0 (5)	C17—C18—C19—C14	−1.1 (4)
C9—C10—C11—C12	1.1 (5)	C15—C14—C19—C18	0.2 (4)
C8—C7—C12—C11	0.0 (4)	C13—C14—C19—C18	179.7 (2)
P1—C7—C12—C11	175.8 (2)		

### Hydrogen-bond geometry (Å, º)

**Table:**

*D*—H···*A*	*D*—H	H···*A*	*D*···*A*	*D*—H···*A*
O2—H2···O1^i^	0.82	1.81	2.613 (2)	168
O4—H4···O3^ii^	0.82	2.28	3.046 (3)	156

Symmetry codes: (i) −*x*+1, −*y*, −*z*+2; (ii) *x*−1/2, −*y*+1/2, *z*−1/2.
